# T-Cell Phenotypes, Apoptosis and Inflammation in HIV+ Patients on Virologically Effective cART with Early Atherosclerosis

**DOI:** 10.1371/journal.pone.0046073

**Published:** 2012-09-27

**Authors:** Esther Merlini, Kety Luzi, Elisa Suardi, Alessandra Barassi, Maddalena Cerrone, Javier Sánchez Martínez, Francesca Bai, Gian Vico Melzi D’Eril, Antonella D’Arminio Monforte, Giulia Marchetti

**Affiliations:** 1 Clinic of Infectious Diseases and Tropical Medicine, Department of Health Sciences, University of Milan, San Paolo Hospital, Milan, Italy; 2 Laboratory of Clinical Analyses, Department of Health Sciences, University of Milan, San Paolo Hospital, Milan, Italy; University of New South Wales, Australia

## Abstract

**Objective:**

We investigated the potential relationship between T-cell phenotype, inflammation, endotoxemia, and atherosclerosis evaluated by carotid intima-media thickness (IMT) in a cohort of HIV-positive patients undergoing long-term virologically suppressive combination antiretroviral therapy (cART).

**Design:**

We studied 163 patients receiving virologically suppressive cART.

**Methods:**

We measured IMT (carotid ultrasound); CD4+/CD8+ T-cell activation (CD38, CD45R0), differentiation (CD127), apoptosis (CD95), and senescence (CD28, CD57) (flow cytometry); plasma sCD14, IL-6, TNF- α, sVCAM-1, hs-CRP, anti-CMV IgG (ELISA); LPS (LAL). The results were compared by Mann-Whitney, Kruskal-Wallis or Chi-square tests, and factors associated with IMT were evaluated by multivariable logistic regression.

**Results:**

Of 163 patients, 112 demonstrated normal IMT (nIMT), whereas 51 (31.3%) had pathological IMT (pIMT: ≥1 mm). Of the patients with pIMT, 22 demonstrated an increased IMT (iIMT), and 29 were shown to have plaques. These patient groups had comparable nadir and current CD4+, VLs and total length of time on cART. Despite similar proportions of CD38-expressing CD8+ cells (p = .95), pIMT patients exhibited higher activated memory CD8+CD38+CD45R0+ cells (p = .038) and apoptotic CD4+CD95+ (p = .01) and CD8+CD95+ cells (p = .003). In comparison to nIMT patients, iIMT patients tended to have lower numbers of early differentiated CD28+CD57− memory CD4+ (p = .048) and CD28–CD57−CD8+ cells (p = .006), both of which are associated with a higher proliferative potential. Despite no differences in plasma LPS levels, pIMT patients showed significantly higher circulating levels of sCD14 than did nIMT patients (p = .046). No differences in anti-CMV IgG was shown. Although circulating levels of sCD14 seemed to be associated with a risk of ATS in an unadjusted analysis, this effect was lost after adjusting for classical cardiovascular predictors.

**Conclusions:**

Despite the provision of full viral suppression by cART, a hyperactivated, pro-apoptotic T-cell profile characterizes HIV-infected patients with early vascular damage, for whom the potential contribution of subclinical endotoxemia and anti-CMV immunity should be investigated further.

## Introduction

HIV-infected patients are at an increased risk for cardiovascular events in comparison to age-matched HIV-negative controls [1], [2]. The reason for this increased risk is multifactorial and involves traditional risk factors, exposure to specific antiretroviral drugs and HIV infection itself [1] [2], [3]. The interaction between HIV infection and cardiovascular disease has been a major concern of the HIV field since the early cART era, when large cohort studies demonstrated a relationship between antiretroviral exposure and myocardial infarction [4]–[11]. Recent studies have introduced the hypothesis that chronic inflammation and immune activation can contribute to the initiation and progression of atherosclerosis (ATS) in the setting of HIV infection [12]–[15]. Recently, some authors have also suggested an association between T-cell activation/senescence and markers of subclinical carotid artery disease, even among patients on stable cART [16].

The role of inflammation and endothelial activation/dysfunction in the development of ATS has been studied extensively in the general population, and several markers, such as sVCAM-1, sICAM-1 and von Willebrand factor antigen, have been shown to reliably indicate the increased activation of endothelial cells in ATS [17], [18]. Tumor necrosis factor (TNF)-α has been implicated in myocardial dysfunction resulting from acute coronary syndrome [19], and high levels of C-reactive protein (CRP) and interleukin (IL)-6 have been associated with subclinical ATS [19]–[21].

In recent years, microbial translocation (MT) has been proposed as a main mechanism behind immune hyperactivation during HIV infection [22]–[25], and recent studies have suggested the potential involvement of MT in the pathogenesis of ATS [26], [27].

The Bruneck study in 1999 provided the first epidemiological evidence in support of a clinical association between levels of lipopolysaccharide (LPS), MT markers, and cardiovascular risk [28]. Very recently, data from the SMART study suggested that high levels of circulating sCD14, a soluble form of the LPS receptor expressed by monocytes, were associated with an increased risk of all-cause mortality, suggesting a link between gut damage, inflammation, immune activation and CD4+ T-cell loss [14].

Long-term successfully treated HIV infected patients have been shown to present remarkably high levels of CMV-specific effector cells, similar to that observed in the elderly [29], allowing to speculate a role of the CMV-specific inflammatory response in immunosenescence and non-AIDS morbidity and mortality. Indeed, Hsue *et al*. demonstrated an independent association between CMV-specific T-cell responses and increased carotid intima-media thickness (IMT) in HIV-infected subjects [30], in keeping with the role of CMV in posttransplant ATS [31]. Most recently, heightened CMV antibody titers were associated with several markers of subclinical ATS in HIV-infected patients on virologically-suppressive cART [32].

As few studies have comprehensively investigated the possible relationship(s) between inflammatory/endothelial activation markers, MT, T-cell immune phenotype, anti-CMV IgG and ATS in HIV-infected individuals upon full HIV-viremia suppression, we conducted this research to assess whether inflammation, endotoxemia and an activated/senescent immune T-cell phenotype would be associated with increased vascular disease, as evaluated by IMT, in HIV-infected patients.

## Materials and Methods

### Study Population

HIV-positive patients were consecutively enrolled at the Clinic of Infectious Diseases at San Paolo Hospital, Milan (Italy), after providing written, informed consent. The study was approved by the Ethical Committee at San Paolo Hospital, Milan (Italy). To be included in the study, patients must have received stable HAART, which was defined as continuous treatment with ≥3 antiretroviral drugs (including either a protease inhibitor or a non-nucleoside reverse transcriptase inhibitor) for at least 6 months, and demonstrated undetectable HIV viremia (<40 cp/mL) at two consecutive assessments. Patients were evaluated for traditional cardiovascular risk factors, and the 10-year risk of acute coronary events was evaluated using the Framingham risk score (FRS) according to the NCEP-ATP-III equation, whereby risk was classified as low (<10%), medium (10–20%) or high (>20%). The homeostasis model assessment of insulin resistance (HOMA-IR) was calculated using the following formula: HOMA-IR = (fasting glucose (mg/dL) × fasting insulin (mU/mL)/405.

### Carotid Artery Ultrasound

IMT measurements were obtained for each patient using a B-mode ultrasound recording with a 7- to 14-MZ array probe (ESAOTE-technology). Patients were placed in the supine position in a dark, quiet room, and the right and left carotid arteries were imaged with the head in the midline position and tilted slightly upwards. The common carotid, the bifurcation and at least the first 2 cm of the internal carotid were examined on the long and short axes. In addition, 3 measurements were made at the far and near walls of each internal carotid and specifically at the carotid bifurcation, the bulb and 1 cm after the bifurcation. The mean value (in mm) of the 3 measurements taken at each site of the internal carotid (left and right) was calculated for each patient and used as the final measurement of internal carotid IMT.

According to published population studies showing that IMT was above 1 mm are significantly associated to a significantly increased hazard of cardiovascular events [33], [34], we defined normal IMT (nIMT) as IMT ≤1 mm and pathological IMT (pIMT) as IMT >1 mm. We further distinguished pIMT as increased IMT (iIMT; for IMT values >1 mm but <1.5 mm) or the presence of a carotid plaque (for IMT values ≥1.5 mm at each site or 50% increased if the near-wall thickness was >1.5 mm). All the measurements of carotid IMT were performed by a single operator to avoid inter-operator differences.

### Evaluation of the T-cell Phenotype

Lymphocyte surface phenotypes were evaluated by flow cytometry using fresh peripheral blood (Coulter ESP; Beckman Coulter, Hialeah, FL) samples stained with the following fluorochrome-labeled antibodies: CD4-Pcy7, CD8-Pcy5, CD38-FITC, CD45-ECD, CD45R0-PE, CD95-FITC and CD127-PE. We evaluated activation (via the expression of CD45R0 and CD38 on CD8+ cells), apoptosis (via the expression of CD95 on CD4+ and CD8+ cells) and IL-7 receptor (CD127) expression on CD8+ and CD4+ T-cells. The following combinations of markers were used: CD8/CD38, CD8/CD38/CD45R0, CD8/CD4/CD95 and CD8/CD4/CD127.

Due to laboratory workflow, T-cell immunosenescence was measured by flow cytometry on cryopreserved PBMCs that had been collected and frozen the same day of fresh cells processing. The following combination of antibodies were used: CD28-PE, CD57-FITC, CD8-PE-Cy5, and CD4-PE-Cy7 (Instrumentation Laboratory, Watertown, Boston, MA, USA). To check cell viability, cells were stained with 7-aminoactynomycin D (7-AAD) for 30 min in dark at 4°C. Only only samples with viability greater than 70% were used for the flow cytometry evaluations.

### Plasma Assays

Plasma levels of sCD14, IL-6, TNF-α and sVCAM-1 were measured by ELISA (R&D) according to the manufacturer’s protocol. We generated a double standard curve for each ELISA plate. We considered reliable only those plates in which the standard curve was comparable to those reported in the manufacturers’ instructions.

The quantification of IgG antibodies against hCMV was performed by a chemioluminescent assay LIAISON CMV IgG II (DiaSorin, Saluggia, Vercelli, Italy) on plasma samples according to manufacturer’s instructions.

### LPS Quantification

Plasma levels of LPS were determined using a commercial LAL kit (Kinetic-QCL; BioWhittaker, Walkersville, MD, USA).

### Statistical Analysis

Continuous variables are expressed as the median and the interquartile range (IQR), and categorical variables are expressed as absolute numbers and percentages. Pro-inflammatory cytokines, MT markers and peripheral T-lymphocyte immune phenotypes in patients with nIMT or pIMT were analyzed using the Mann-Whitney U test. Differences between patients with nIMT, iIMT and plaques were assessed using the Kruskal-Wallis and Chi-square tests for continuous and categorical variables, respectively. Peripheral immune parameters that yielded a p value <.05 with the Kruskal-Wallis test were further analyzed using the Mann-Whitney U test to compare the nIMT vs. iIMT patients, nIMT vs. plaque patients and iIMT vs. plaque patients.

To explore the factors independently associated with pIMT, considered as a categorical variable, we used two multivariate logistic regression models. In the first model, we assessed the association between sCD14 levels and IMT, adjusting for FRS and HOMA-IR. In the second one, we included the following variables: FRS, PI exposure, HOMA-IR, CD4+ cell count, sCD14 levels, and the frequencies of CD8+CD38+, CD8+CD45R0+, CD4+CD95+ and CD8+CD127+ cells. We then performed two linear regression models, considering the outcome as a continuous parameter, i.e. mean IMT, including the same variables used for logistic regression analysis.

All analyses were performed using SPSS (version 18.01), and p values ≤.05 were considered statistically significant.

## Results

### Patient Characteristics

Of the 163 patients enrolled in the study, 112 were found to have nIMT, 22 demonstrated iIMT, and 29 were found to have carotid plaques. The definition of pIMT was used when patients with iIMT and/or plaques were grouped together. The demographic, clinical, and HIV-related characteristics as well as fasting metabolic parameters according to IMT group are shown in Table 1.

**Table 1:** Patients’ characteristics.

	Patients	nIMT	iIMT	Plaque	p
	n = 163	n = 112	n = 22	n = 29	
Age (years)*	48 (43–54)	46 (42–65)	55 (46–68)	50 (47–61)	0.0001
Sex (male) °	134 (82)	90 (80)	20 (91)	23 (79)	0.476
Caucasian °	154 (94)	104 (93)	22 (100)	27 (93)	0.668
Current IDUs °	47 (29)	33 (29)	5 (23)	9 (21)	0.776
HCV Ab °	46 (28)	28 (25)	6 (27)	12 (41)	0.216
HCV-RNA (IU/mL)*	754400 (17–2893000)	461827 (3823–21890000)	619701 (17–5305000)	1842000 (14–3258000)	0.707
Cirrhosis (yes)°	10 (6)	5 (4)	1 (4)	4 (13)	0.522
**HIV-related parameters**					
Years since HIV diagnosis*	12 (5–19)	11.5 (5–19.7)	14 (8.7–19.2)	11 (5–19)	0.79
HAART°					0.523
*NNRTI*	56 (34)	42 (37)	6 (27)	8 (27)	
*PI*	91 (56)	61 (55)	12 (55)	18 (62)	
*Other*	16 (10)	9 (8)	4 (18)	3 (11)	
Total years of HAART*	5 (2–11)	4 (2–10)	7 (3–9)	5 (2–13)	0.725
NNRTI duration, (months)*	56.2 (16.6–96.5)	44 (17–114)	71 (39–119)	59 (22–158)	0.392
NRTI duration, (months)*	53.3 (18.9–128.7)	50 (15–95)	59 (10–85)	59 (27–109)	0.525
PI duration, (months)*	22.7 (12.9–111.6)	20 (11–108)	50 (19–56)	29 (14–132)	0.648
Nadir CD4+ T-cells	210 (99–326)	216 (119–336)	163 (50–308)	198 (28–387)	0.245
(cells/uL)*					
Current CD4+ T-(cells/uL)*	496 (358–718)	500 (362–708)	465 (328–577)	512 (341–765)	0.736
Delta CD4+ T-cells	276 (139–448)	276 (128.5–435)	258 (168–521)	266 (167.2–395)	0.87
(cell/uL)*					
Current HIV RNA	1.59	1.59	1.59	1.59	0.18
(Log _10 _cp/mL)*					
**Cardiovascular risk factors**					
Current smoking°	78 (48)	54 (48)	11 (50)	13 (45)	0.919
Hypertension °	26 (16)	10 (9)	8 (36)	8 (27)	0.001
History of Coronary Disease °	8 (5)	4 (4)	2 (9)	2 (7)	0.473
BMI*	24.6 (22.9–26.7)	25 (23–26)	25 (22–27)	25 (23–27)	0.828
Framingham Score*	6 (2.2–11)	4 (2–9)	11 (6–16)	8 (4.5–16)	0.0001
**Laboratory Values**					
Total Cholesterol, (mg/dL)*	190.5 (159–220)	192 (159–221)	168 (142–208)	192 (173–208)	0.44
LDL Cholesterol, (mg/dL)*	111 (83–136.2)	114 (86–138)	89 (71–137)	112 (92–131)	0.564
HDL Cholesterol, (mg/dL)*	43 (35–54)	44 (35–54)	37 (31–48)	42 (36–52)	0.274
Triglycerides, (mg/dL)*	140 (160.2–194)	137 (104–194)	143 (110–211)	158 (112–178)	0.827
Fasting glucose, (mg/dL)*	96 (88–104)	95 (87–102)	99 (89–113)	96 (88–109)	0.185
HOMA-IR*	2.6 (1.6–4.6)	2.2 (1.4–3.9)	5 (1.5–12.3)	3.5 (2.9–7.9)	0.001
Lipid-lowering therapy°	40 (24)	23 (21)	7 (32)	10 (34)	0.219

NOTE: nIMT, normal intima-media thickness (IMT) ≤1 mm; iIMT, increased IMT (>1 mm and <1.5 mm); Plaque, IMT ≥1.5 mm at each site or a 50% increase for near-wall thickness >1.5 mm; IDUs, intravenous drug users; HAART, highly active antiretroviral therapy; NNRTI, non-nucleoside reverse transcriptase inhibitors; NRTI, nucleoside reverse transcriptase inhibitors; PI, protease inhibitor; Delta CD4, current CD4+ T-cell count – nadir CD4 T-cell count; LDL, low-density lipoprotein; HDL, high-density lipoprotein; BMI, body mass index; HOMA, homeostatic model assessment index.

* Data are presented as medians (interquartile range). Kruskal-Wallis test.

°Data are presented as absolute numbers (percentages). Pearson’s Chi-square test.

The patient groups were similar with respect to race, sex and body mass index (BMI), although patients with nIMT had a significantly lower median age (p = .0001). Traditional cardiovascular risk factors, such as current smoking status and a history of coronary disease, were similar between the three patients groups, although hypertension and FRS were significantly lower among nIMT patients (p = .001 and p = .0001, respectively). There were also no differences between groups in terms of cholesterol (HDL and LDL), triglyceride and plasma glucose levels, although the HOMA-IR values were significantly higher among iIMT patients (p = .001).

All participants had undetectable plasma HIV RNA levels. The median (IQR) CD4+ T-cell count was 497/mmc (IQR: 358–718), and there were no significant differences between groups.

28% of our patients’ cohort was HCV Ab positive. However, no significant differences in HCV RNA levels and presence of liver chirrosis were shown between groups.

### Immunophenotypic Markers of T-cell Activation, Apoptosis and Differentiation According to Carotid Intima-media Thickness

**Figure 1:**
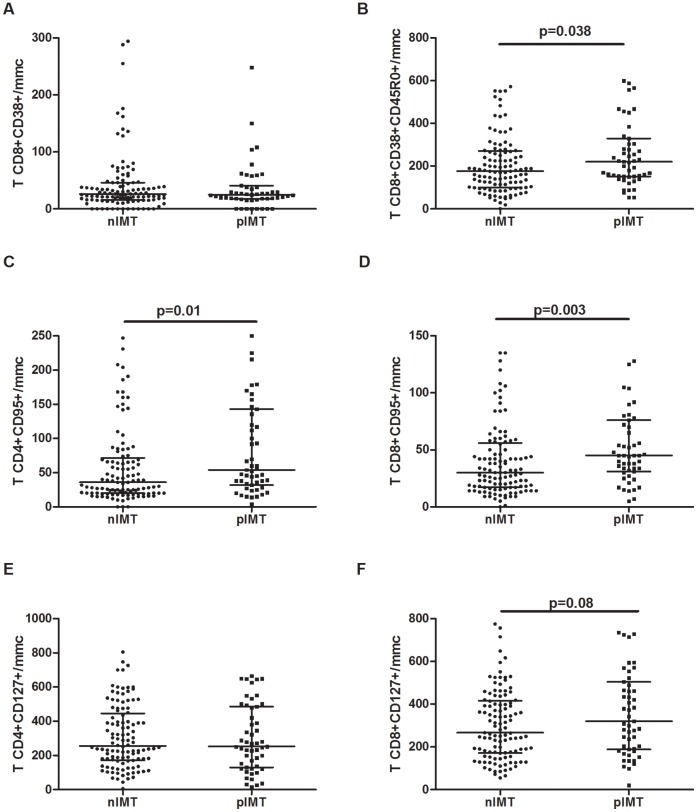
Different peripheral T-cell immune phenotypes according to the degree of carotid intima-media thickness. **A–B.** Activated CD8+ T-cells were defined by the expression of CD38, whereas memory activated CD8+ T-cells were defined by the co-expression of CD45R0 and CD38. **A.** nIMT and pIMT HIV+ patients exhibited similar number of CD8+CD38+ T-cells. **B.** pIMT patients had significantly higher memory activated CD8+CD38+CD45R0+ T-cells in comparison to nIMT patients (p = .038). **C–D.** Apoptotic T-cells were defined by the expression of CD95 on CD4+ and CD8+ cells. As compared to nIMT, pIMT patients exhibited a significantly higher number of CD4+CD95+ cells (p = .01) (**C**), and CD8+CD95+ T-cells (p = .003) (**D**). **E.** CD127 expression on CD4+ T-cells was similar between the nIMT and pIMT groups. **F.** A non-significant trend towards greater number of CD8+CD127+ cells was observed among pIMT patients as compared to nIMT patients (p = .08).

In comparison to HIV-positive patients with nIMT, patients with pIMT exhibited a similar number of CD38-expressing CD8+ T-cells (nIMT 25/mmc [IQR: 15–43] vs. pIMT 25/mmc [IQR: 18–41], p = .95; Fig. 1A), and there was a similar trend even when the pIMT patients were divided into those with iIMT or plaques (iIMT 25/mmc [IQR: 18–60] vs. plaque 24/mmc [IQR: 16–38], p = .80 for the comparison between nIMT, iIMT and plaque).

Interestingly, pIMT patients had a significantly higher number of activated memory CD8+CD38+CD45R0+ T-cells than nIMT patients (221/mmc [IQR: 152–330] vs. 176/mmc [IQR: 100–272], p = .038; Fig. 1B). A similar trend was also observed when nIMT patients were compared to patients with iIMT or plaques (p = .08 for the comparison between nIMT, iIMT and plaque patients), and significant differences were only found between patients with nIMT and plaques (plaque 225/mmc [IQR: 153–317]; p = .029 for nIMT vs. plaque; p = .23 for nIMT vs. iIMT). Patients with plaques or iIMT exhibited comparable number of CD8+CD38+CD45R0+ cells (iIMT 199/mmc [IQR: 113–160], p = .56).

Regarding CD95 expression, pIMT patients exhibited significantly higher number of CD4+CD95+ (54/mmc [IQR: 32–143] vs. 36/mmc [IQR: 20–72], p = .01; Fig. 1C) and CD8+CD95+ T-cells (45/mmc [IQR: 31–76] vs. 30/mmc [IQR: 17–53], p = .003; Fig. 1D) in comparison to nIMT patients. In particular, the number of CD4+CD95+ T-cells was significantly higher among both patients with iIMT and plaques than those with nIMT (iIMT 63/mmc [IQR: 30–151], p = .028; plaque 48/mmc [IQR: 31–126], p = .07), and there were no differences between the iIMT and plaque patients (p = .70). Conversely, only plaque patients exhibited significantly increased frequencies of CD8+CD95+ T-cells than nIMT patients (iIMT 37/mmc [IQR: 26–67]; plaque 48/mmc [IQR: 36–79]; p = .21 and p = .002, respectively; p = .14 for iIMT vs. plaque).

The number of CD127-expressing CD4+ T-cells was similar between the nIMT and pIMT groups (nIMT 259/mmc [IQR: 171–450] vs. pIMT 252/mmc [IQR: 130–486], p = .77; Fig. 1E), and this similarity persisted even when compared across the three study groups (iIMT 251/mmc [IQR: 124–356]; plaque 272/mmc [IQR: 141–518]; p = .83 for the comparison between nIMT, iIMT and plaque). A non-significant trend towards higher levels of CD127-expressing CD8+ T-cells was detected for the nIMT and pIMT groups (nIMT 265/mmc [IQR: 171–417] vs. pIMT 320/mmc [IQR: 188–504], p = .08; Fig. 1F), although no differences were found after dividing the pIMT patients into the iIMT and plaque groups (iIMT 292/mmc [IQR: 177–432]; plaque 379/mmc [IQR: 197–570]; p = .15 for the comparison between nIMT, iIMT and plaque).

Data on T-cell immunephenotypes frequencies are presented in Supplementary Table 1.

### Immunophenotypic Markers of T-cell Senescence According to Carotid Intima-media Thickness

**Figure 2:**
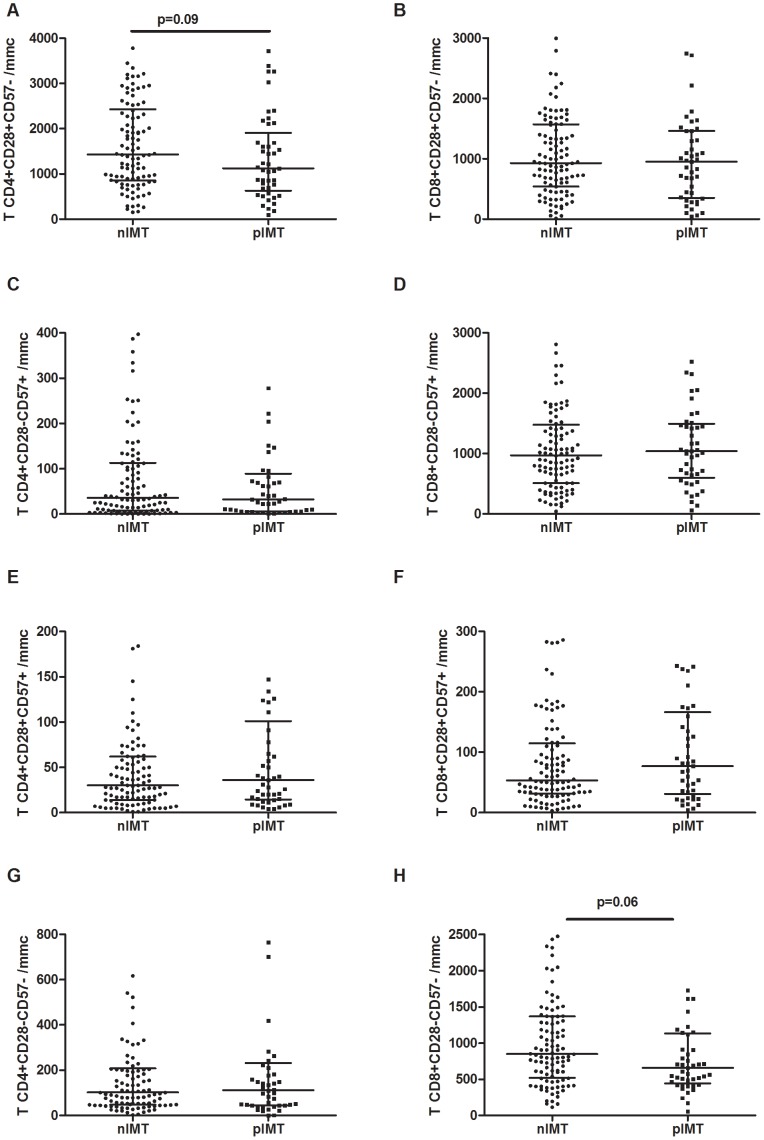
T-cell immunosenescence according to the degree of intima-media thickness. **A.** A non-significant tendency towards reduced early differentiated memory (CD28+CD57−) CD4+ T-cell numbers was observed for pIMT patients in comparison to nIMT patients (p = .09). **B.** No differences were observed in early differentiated memory CD8+ CD28+CD57− T-cells between the two study groups. **C–D.** The number of late-differentiated memory (CD28–CD57+) CD4+ (**C**) and CD8+ (**D**) T-cells was comparable between nIMT and pIMT groups. **E–F.** We observed no difference in CD4+CD28+CD57+ (**E**) and CD8+CD28+CD57+ (**F**) T-cells between the nIMT and pIMT groups. **G.** No major difference in CD4+CD28–CD57− T-cells were observed between nIMT and pIMT patients. **H.** Compared to nIMT patients, pIMT patients tended to have lower number of CD8+CD28–CD57− cells (p = .06).

Immunophenotypic markers of T-cell senescence were measured in a randomly selected subgroup of 151 patients. A non-significant tendency towards lower levels of early differentiated memory CD4+CD28+CD57− T-cells among pIMT patients as compared to nIMT patients was shown (1124/mmc [IQR: 629–1906] vs. 1431/mmc [IQR: 856–2429], p = .09; Fig. 2A). Similarly, lower CD4+CD28+CD57− T-cells persisted when the pIMT patients were divided into the iIMT and plaque groups (iIMT 1146/mmc [IQR: 577–1608]; plaque 1105/mmc [IQR: 662–2275]), and this difference reached statistical significance only for iIMT patients (p = .048 and p = .13 for iIMT and plaque vs. nIMT, respectively; p = .35 for iIMT vs. plaque). No differences were observed in CD8+CD28+CD57− between the nIMT and pIMT groups (930/mmc [IQR: 545–1571] vs. 954/mmc [IQR: 354–1463], p = .45; Fig. 2B; iIMT 1017/mmc [IQR: 283–1314]; plaque 848 [IQR: 419–1510]; p = .74 for the comparison between nIMT, iIMT and plaque).

The number of late-differentiated memory CD4+CD28–CD57+ T-cells was comparable between the nIMT and pIMT groups (nIMT 32/mmc [IQR: 8–113] vs. pIMT 32/mmc [IQR: 6–89], p = .92; Fig. 2C; iIMT 41/mmc [IQR: 6–147] vs. plaque 30/mmc [IQR: 7–70], p = .71 for the comparison between nIMT, iIMT and plaque). Accordingly, the pIMT group exhibited similar CD8+CD28–CD57+ T-cell number (nIMT 975/mmc [IQR: 511–1479] vs. pIMT 1040/mmc [IQR: 601–1495], p = .68; Fig. 2D; iIMT 1000/mmc [IQR: 512–1475] vs. plaque 1042/mmc [IQR: 676–1519], p = .88 for the comparison between nIMT, iIMT and plaque;).

We observed no differences in CD4+CD28+CD57+ T-cells between nIMT and pIMT patients (30/mmc [IQR: 14–62] vs. 36/mmc [IQR: 15–101], p = .44; Fig. 2E; iIMT 31/mmc [IQR: 12–111] vs. plaque 37/mmc [IQR: 15–89], p = .74 for the comparison between nIMT, iIMT and plaque). Accordingly, the number of CD28/CD57-co-expressing CD8+ T-cells was similar among both nIMT and pIMT patients (54/mmc [IQR: 31–115] vs. 77/mmc [IQR: 31–166], p = .28; Fig. 2F; iIMT 82/mmc [IQR: 36–235] vs. plaque 69/mmc [IQR: 23–145], p = .34 for the comparison between nIMT, iIMT and plaque).

No major differences were shown in CD4+CD28–CD57− T-cells between nIMT and pIMT patients (102/mmc [IQR: 48–209] vs. 112/mmc [IQR: 45–233], p = .94; Fig. 2G; iIMT 112/mmc [IQR: 45–176] vs. plaque 116/mmc [IQR: 43–302], p = .86 for the comparison between nIMT, iIMT and plaque).

Interestingly, we found differences within the CD8+CD28–CD57− T-cell subset. In particular, as compared to nIMT patients, pIMT patients had a non-significant tendency towards reduced CD8+CD28–CD57− cell numbers (nIMT 848/mmc [IQR: 521–1367] vs. pIMT 660/mmc [IQR: 442–1132], p = .06; Fig. 2H). Moreover, this difference retained statistical significance after the pIMT group was divided into iIMT and plaque patients (iIMT 524/mmc [IQR: 404–708]; plaque 771/mmc [IQR: 504–1278]; p = .02 for the comparison between nIMT, iIMT and plaque; nIMT vs. iIMT, p = .006). Interestingly, patients with plaques demonstrated slightly increased IMT values as compared to iIMT patients (p = .03).

Data on T-cell immunephenotypes frequencies are presented in Supplementary Table 1.

### Soluble Markers of Inflammation and Endothelial Cell Activation According to Carotid Intima-media Thickness

**Figure 3:**
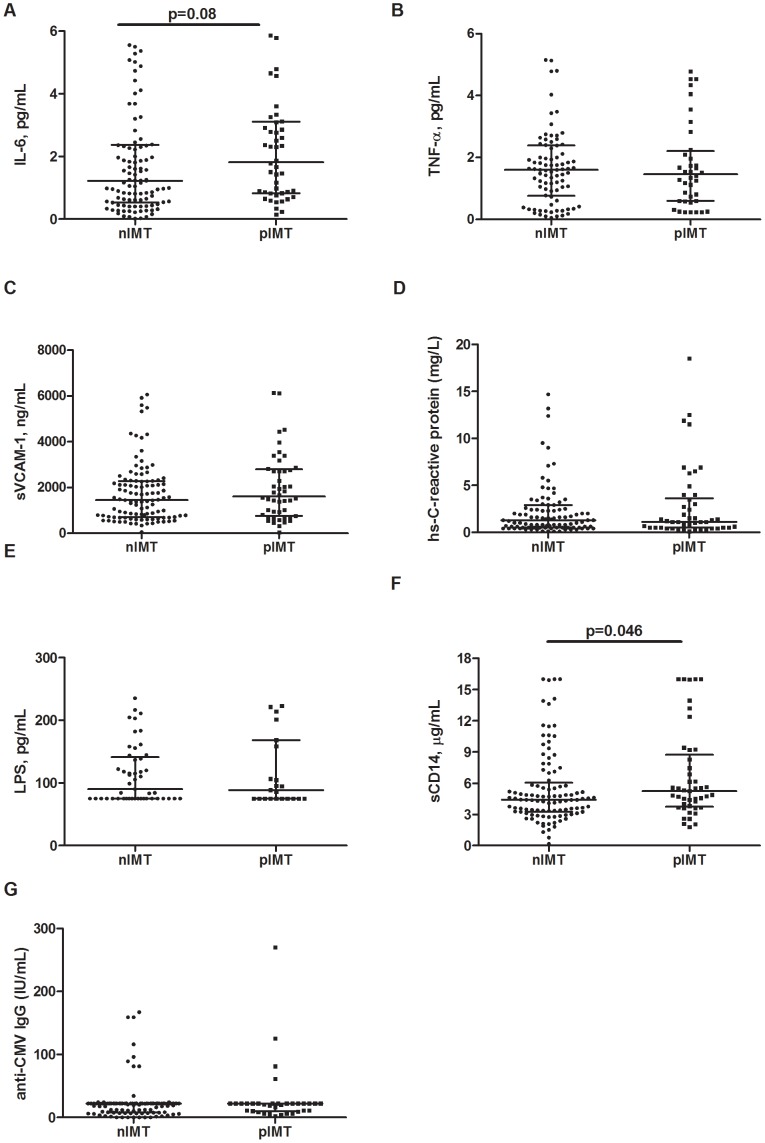
Markers of Inflammation, endothelial cell activation, microbial translocation and anti-CMV IgG according to the degree of intima-media thickness. **A.** IL-6 plasma levels were increased in pIMT patients in comparison to nIMT patients, albeit not-reaching significance (p = .08). **B–F.** When nIMT patients were compared to pIMT patients, no differences in TNF-α (**B**), s-VCAM-1 (**C**) hs-C-reactive protein (hs-CRP) (**D**) plasma levels were detected. **E.** nIMT and pIMT patients exhibited similar plasma levels of lipopolysaccharide (LPS). **F.** pIMT patients showed significantly higher circulating levels of sCD14 in comparison to nIMT patients (p = .046). **G.** nIMT and pIMT patients displayed comparable levels of anti-CMV IgG.

A non-significant tendency towards higher IL-6 plasma levels was found among pIMT patients as compared to nIMT patients (1.67 pg/mL [IQR: 0.82–3.96] vs. 1.23 pg/mL [IQR: 0.56–2.37], p = .08; Fig. 3A), but this increase was lost when the pIMT group was divided into iIMT and plaque patients (iIMT 1.48 pg/mL [IQR: 0.59–3.69]; plaque 1.78 pg/mL [IQR: 0.87–4.58]; p = .18 for the comparison between nIMT, iIMT and plaque).

No differences in the plasma levels of TNF-α were observed when we compared either nIMT patients to pIMT patients (1.58 pg/mL [IQR: 0.67–2.39] vs. 1.51 pg/mL [IQR: 0.65–2.07], p = .96; Fig. 3B) or nIMT patients to iIMT and plaque patients (iIMT 1.20 pg/mL [IQR: 0.45–1.89]; plaque 1.67 pg/mL [IQR: 0.87–2.84]; p = .49 for the comparison between nIMT, iIMT and plaque).

Circulating s-VCAM-1 levels in pIMT patients were similar to those in nIMT patients (nIMT 1522 ng/mL [IQR: 687–2286] vs. pIMT 1476 ng/mL [IQR: 857–2707], p = .39; Fig. 3C). Moreover, no differences were observed when the HIV+ patients were separated into the three analysis groups (iIMT 1540 ng/mL [IQR: 764–2438]; plaque 1432 ng/mL [IQR: 995–2810]; p = .49 for the comparison between nIMT, iIMT and plaque).

Hs-C-reactive protein (hs-CRP) levels were similar between normal IMT patients and patients with pathological IMTs (nIMT 1.4 mg/L [IQR: 0.5–3] vs. pIMT 1.1 mg/L [IQR: 0.5–3.25], p = .41; Fig. 3D). Moreover, the circulating levels of hs-CRP were comparable even following the division of patients into the nIMT, iIMT and plaque groups (iIMT 0.7 mg/L [IQR: 0.5–1.7]; plaque 1.3 mg/L [IQR: 0.4–4.7]; p = .37 for the comparison between nIMT, iIMT and plaque).

### Markers of Microbial Translocation and CMV IgG According to Carotid Intima-media Thickness

nIMT and pIMT patients exhibited similar plasma levels of LPS (nIMT 94 pg/mL [75–142] vs. pIMT 80 pg/mL [IQR: 75–145], p = .72; Fig. 3E). In addition, the levels of LPS remained comparable after the patients were divided into the iIMT and plaque groups (iIMT 100 pg/mL [IQR: 83–206]; plaque 75 pg/mL [IQR: 75–111]; p = .19 for the comparison between nIMT, iIMT and plaque).

Interestingly, despite the lack of differences in plasma LPS levels, the pIMT group exhibited significantly higher circulating levels of sCD14 as compared to the nIMT group (5.19 µg/mL [IQR: 3.85–9.09] vs. 4.41 µg/mL [IQR: 3.32–5.79], p = .046; Fig. 3F). However, when we analyzed the iIMT and plaque patients separately, this difference was lost (iIMT 5.59 µg/mL [IQR: 3.66–9.24]; plaque 4.86 µg/mL [IQR: 3.96–8.91]; p = .12 for the comparison between nIMT, iIMT and plaque).

87% of our patients resulted anti-CMV positive. However, no significant differences were shown in anti-CMV IgG titer neither comparing nIMT *versus* pIMT (22 [10]–[22] IU/ml vs 22 [10]–[22] p = .0.86), nor comparing nIMT, iIMT and plaque (22 [10]–[22] IU/ml vs 22 [16]–[22] IU/ml vs 19 [8]–[22] IU/ml; p = .57). (Figure 3G). No significant association was shown between anti-CMV IgG titer, CD8+CD38+CD45R0+ (Rho = −0.052, p = 0.592) and CD4+/CD8+CD95+ T-cells (Rho = 0.053, p = 0.589; Rho = 0.061, p = 0.534, respectively). Interestingly enough, when the correlation analysis was performed only in patients with pathological IMT (pIMT), a slight positive correlation was shown between anti-CMV IgG titer and pro-apoptotic CD4+CD95+ T-cells (Rho = 0.41, p = 0.0136).

### Identification of Factors Associated with Carotid Intima-media Thickness by Univariate and Multivariate Analyses

Traditional risk factors and immunological or soluble markers that displayed a p value <.01 for the Mann-Whitney U test were included in a logistic regression model to investigate the independent factors associated with increased IMT and/or plaques, as shown in Table 2a. Given the integrative nature of FRS and HOMA-IR that altogether include several traditional cardiovascular risk factors, for multivariate models we specifically chose not to include other risk factors that were not associated in the univariate models.

**Table 2:** Regression models to explore independent factors associated with intima-media thickness (pIMT).

Table 2a	Univariate			Multivariate*			Multivariate**		
	Beta	95%CI	p	Beta	95%CI	p	Beta	95%CI	p
Framingham risk score	1.137	1.070–1.208	**0.0001**	1.133	1.055–1.216	**0.001**	1.134	1.052–1.222	**0.001**
(each unit more)									
PI exposure	1.054	0.978–1.137	0.169				0.986	0.884–1.099	0.795
(each year more)									
HOMA-IR	1.170	1.075–1.274	**0.0001**	1.150	1.048–1.261	**0.003**	1.146	1.041–1.261	**0.005**
(each unit more)									
CD4+T-cell count	1.000	0.998–1.001	0.694				1.000	0.998–1.002	0.761
(each cell/mmc more)									
sCD14 ug/mL	1.097	1.003–1.198	**0.042**	1.089	0.981–1.208	0.110	1.085	0.969–1.215	0.156
(each unit more)									
CD8+CD38+ %	0.992	0.941–1.045	0.755				1.037	0.953–1.128	0.404
(each unit more)									
CD8+CD45R0+CD38+ %	0.997	0.978–1.017	0.791				0.992	0.965–1.019	0.549
(each unit more)									
CD4+CD95+ %	1.085	0.998–1.179	**0.055**				1.060	0.952–1.181	0.285
(each unit more)									
CD8+CD127+ %	1.025	0.980–1.072	0.275				1.020	0.959–1.084	0.528
(each unit more)									
**Table 2b**	**Univariate**			**Multivariate** *			**Multivariate** **		
	**Beta**	**95%CI**	**p**	**Beta**	**95%CI**	**p**	**Beta**	**95%CI**	**p**
Framingham risk score	0.322	0.006–0.018	**0.0001**	0.336	0.006–0.012	**0.0001**	0.312	0.006–0.018	**0.0001**
(each unit more)									
PI exposure	0.186	0.002–0.018	**0.018**				0.096	−0.003–0.014	0.239
(each year more)									
HOMA-IR	0.271	0.006–0.021	**0.001**	0.211	0.003–0.018	**0.008**	0.190	0.002–0.017	**0.018**
(each unit more)									
CD4+T-cell count	−0.111	0.000–0.000	0.166				−0.048	0.000–0.000	0.587
(each cell/mmc more)									
sCD14 ug/mL	0.108	−0.003–0.016	0.197	0.056	−0.006–0.012	0.478	0.042	−0.007–0.012	0.608
(each unit more)									
CD8+CD38+ %	0.028	−0.005–0.007	0.729				0.149	−0.001–0.018	0.064
(each unit more)									
CD8+CD45R0+CD38+ %	−0.028	−0.002–0.002	0.727				−0.115	−0.003–0.000	0.105
(each unit more)									
CD4+CD95+ %	0.224	0.004–0.022	**0.005**				0.130	−0.002–0.016	0.105
(each unit more)									
CD8+CD127+ %	−0.025	−0.006–0.004	0.758				−0.072	−0.007–0.003	0.357
(each unit more)									

a Logistic regression model: pathological intima-media thickness (pIMT) defined as IMT>1 mm analysed as categorical variable.

b Linear regression model: mean IMT analysed as a continuous variable –plaque excluded from the analysis.

NOTE: HAART, highly active antiretroviral therapy; PI, protease inhibitors; HOMA-IR, homeostasis model assessment of insulin resistance.

OR, odds ratio – AOR, adjusted odds ratio.

* Adjusted for Framingham risk score and HOMA-IR;

** Mutually adjusted for all of the parameters tested in the univariate model.

The univariate model revealed a significant association between pathological IMT (iIMT and/or plaques) and FRS (OR = 1.137; confidence interval = 1.070, 1.208; p = .0001) and HOMA-IR (OR = 1.170; confidence interval = 1.075, 1.274; p = 0.0001). Interestingly, of the immunologic variables, sCD14 levels (OR = 1.097; confidence interval = 1.003, 1.198; p = .042) and CD4+CD95+ T-cell percentages (OR = 1.085; confidence interval = 0.998, 1.179; p = .055) were associated with pIMT.

However, when these data were analyzed by multivariable logistic regression, only FRS (AOR = 1.134; confidence interval = 1.152, 1.222; p = .001) and HOMA-IR (AOR = 1.146; confidence interval = 1.041, 1.261; p = .005) were confirmed to be independently associated with pathological IMT (Table 2a).

Most interestingly, comparable results were obtained when we performed uni- and multivariate linear regressions analyzing IMT as a continuous variable as shown in Table 2b. While in the univariate model FRS, HOMA-IR, PI exposure, and CD4+CD95+ T-cells were all seemingly associated to increased IMT, only FRS and HOMA-IR were confirmed independently associated by multivariate regression (Table 2b).

## Discussion

In the current study, we evaluated T-cell phenotype, inflammatory biomarkers, microbial translocation, and CMV IgG levels in a cohort of HIV-infected patients receiving cART who demonstrated long-term control of HIV replication, and the analyses were performed according to the degree of ATS measured by internal carotid IMT.

Patients with early ATS were characterized as having a circulating T-cell phenotype dominated by activated memory CD38+CD45R0+ CD8+ cells and apoptosis-committed CD95+ cells and reduced CD57-negative cells, which altogether suggest the greater replicative history of these T-cells. Although higher levels of sCD14 displayed a non-significant association with pIMT, no markers of T-cell activation, inflammation or microbial translocation were able to predict early ATS independent of the classical cardiovascular risk factors.

Our research shows that although cART provides full suppression of HIV viremia, HIV-infected patients with atherosclerotic lesions exhibit a disproportionate expansion of activated memory CD8+ T-cells. The expansion of CD38+CD45R0+ cells, despite the equal proportions of CD8+CD38+ T-cells, may be indicative of increased T-lymphocyte replicative history in a setting of controlled HIV-driven immune activation by effective long-term antiretroviral therapy [35]–[37].

Having shown that the presence of a memory T-cell subset expressing terminal differentiation markers correlated with accelerated ATS, we sought to verify T-cell replicative history by investigating surface CD57/CD28 expression profiles [16], [38]–[41]. Whereas many studies have suggested that CD28 and CD57 expression are mutually exclusive in human T-cells [42], [43], Brenchley *et al.* [44] described both circulating CD28+CD57+ and CD28–CD57− T-cell subpopulations in HIV-positive patients that demonstrated different proliferative histories/potential. We found reduced circulating T-cells lacking CD57 expression (either co-expressing or not co-expressing CD28) in patients with pIMT. Given that the expression of CD57 has been shown to indicate T-cell senescence, the lack of CD57 surface expression therefore defines subsets that have undergone fewer rounds of cell division and have been suggested to posess greater proliferative potential [44], [45].

Therefore, our findings would suggest that patients with subclinical ATS possess a circulating T-cell phenotype impoverished of T-cell subsets with greater proliferative potential. However, these results would suggest that higher levels of antigen-experienced (CD57+) T-cells accompanying IMT represent the counterpart to the reduced proportion of less mature CD57−negative T-cells. In contrast to recently published data [16], we did not find differences in the CD57+ T-cells according to the degree of IMT. Increased peripheral apoptosis within the CD57+ terminally differentiated T-cell subset may represent one potential explanation for this finding, given the higher susceptibility of CD57+ T-cells to activation-induced cell death by apoptosis [44].

Moreover, our patients with pathological IMT exhibited an increased prevalence of apoptosis-committed T-cells expressing the death receptor Fas (CD95) [46].

However, a broader investigation of the correlates of immune activation/inflammation during early vascular damage in HIV-infected patients receiving virologically suppressive cART failed to detect relevant differences in pro-inflammatory biomarkers and the endothelial adhesion marker sVCAM-1, with the exception of a non-significant trend towards higher circulating levels of IL-6 in patients with ATS. This finding is in contrast to previous studies showing that elevated levels of inflammatory markers, such as hs-CRP, are associated with cardiovascular diseases and all-cause mortality in HIV-positive patients [12], [15], [20]. These findings may be secondary to the full suppression of HIV viremia in our cohort, as previous studies have demonstrated an association between inflammatory biomarkers and HIV RNA levels [47].

To investigate the mechanisms governing T-cell activation/senescence in patients with early ATS, we evaluated the levels of LPS and sCD14 in the serum as markers of MT and LPS bioactivity, respectively [22], [23], given the relationship between endotoxemia and cardiovascular disease [27], [28], [48], [49]. Notably, patients with pathological IMT exhibited heightened circulating levels of sCD14 despite normal levels of plasma LPS. Sandler *et al*. recently demonstrated an independent association between high sCD14 levels during HIV infection and an increased risk of all-cause mortality [14]. Although stimulation of monocytes with microbial products stimulates the release of IL-6 and TNF-α, the levels of these cytokines have been shown to be weakly correlated with those of sCD14 and are rather more strongly associated with direct viral factors; this may explain why we did not observe major changes in the levels of IL-6/TNF-α in our virologically suppressed cohort in the context of higher levels of sCD14 [50].

Although circulating levels of sCD14 seemed associated with a risk of ATS in the univariable model, this effect was lost after adjustments were made for classical cardiovascular disease predictors. As a result, only these traditional cardiovascular risk factors were confirmed to independently predict a risk of vascular disease [1], [51], [52].

Opposite to what recently shown by Parrinello et al. in a cohort of HIV-infected treated/aviremic women [32], we failed to find any significant association between IMT and CMV IgG titer. Different duration of both HIV and CMV infection, as well as diverse time on cART could explain the discrepancies between our findings and previous data.

Quite interestingly, in line with recent findings associating CMV IgG levels and markers of immunosenescence [53], our data demonstrated a positive association between anti-CMV response and the pro-apoptotic CD95+ CD4+ T-cell compartment in patients with early signs of ATS, lending support to the role of CMV in accelerating immunosenescence.

The primary limitation of our study is its cross-sectional design, with the lack of a matched control group of HIV-uninfected individuals. Data presented herein would certainly gain much more strength if the immune differences observed according to IMT were not found in a well-matched cohort of HIV-negative individuals, providing stronger evidence for the role of immunological/inflammatory markers in accelerating the early HIV-associated atherosclerosis. Similarly, a control group of matched HIV-infected but antiretroviral-naïve patients would have helped to discriminate between the effect of HIV itself and any potential negative or positive roles played by the introduction of HAART. Furthermore, despite well-balanced patient groups, our study suffers from a relatively small sample size that may have affected the strength of the associations between ATS and the biomarkers investigated.

Whether or not markers of inflammation/immune activation indeed condition the onset of subclinical atherosclerosis independently of traditional cardiovascular risk factors, as well as the possible role of host responsiveness to ongoing endotoxemia and immunity to CMV on vascular damage in cART-suppressed HIV-infected individuals should be further investigated in studies with larger sample sizes.

## Supporting Information

Table S1 **Frequencies of T-cell immunophenotypes according to IMT.** Data are shown as median (IQR, Interquartile Range); p* data analyzed by Kruskal-Wallis for comparison between the 3 groups; p** Mann-Whitney test for comparison between 2 groups; IMT: Intima-Media Thickness; nIMT, normal intima-media thickness (IMT) ≤1 mm; iIMT, increased IMT (>1 mm and <1.5 mm); Plaque, IMT ≥1.5 mm at each site or a 50% increase for near-wall thickness >1.5 mm.
(DOC)
