# Supplementary material for: T-Cell Phenotypes, Apoptosis and Inflammation in HIV+ Patients on Virologically Effective cART with Early Atherosclerosis
Source: PLoS One. 2012 Sep 27;7(9):e46073. doi: 10.1371/journal.pone.0046073 (PMC3459872; doi:10.1371/journal.pone.0046073)
Supplement: Table S1 — Frequencies of T-cell immunophenotypes according to IMT. Data are shown as median (IQR, Interquartile Range); p* data analyzed by Kruskal-Wallis for comparison between the 3 groups; p** Mann-Whitney test for comparison between 2 groups; IMT: Intima-Media Thickness; nIMT, normal intima-media thickness (IMT) ≤1 mm; iIMT, increased IMT (>1 mm and <1.5 mm); Plaque, IMT ≥1.5 mm at each site or a 50% increase for near-wall thickness >1.5 mm. (DOC) [file pone.0046073.s001.doc]

**Table S1. Frequencies of T-cell immunophenotypes according to IMT**

| **T-cell Immunophenotypes (%)** | **nIMT (n=112)** | **iIMT (n=22)** | **Plaque (n=29)** | **P*** | **P**** |
| --- | --- | --- | --- | --- | --- |
| **CD8+CD38+** | 1 (1-2) | 1 (1-3) | 1 (1-2) | 0.269 |  |
| **CD8+CD45R0+CD38+** | 9 (6-15) | 11 (6-16) | 11 (8-17) | 0.281 |  |
| **CD4+CD95+** | 2 (1-4) | 2 (1-9) | 3 (1-6) | 0.146 |  |
| **CD8+CD95+** | 2 (1-3) | 2 (1-4) | 2 (2-4) | **0.006** | **0.008**  nIMT vs plaque |
| **CD4+CD127+** | 16 (9-22) | 13 (8-19) | 16 (10-23) | 0.562 |  |
| **CD8+CD127+** | 15 (10-20) | 14 (10-24) | 17 (9-22) | 0.734 |  |
| **CD4+CD28+CD57-** | 87 (72-94) | 83 (51-91) | 84 (64-93) | 0.493 |  |
| **CD8+CD28+CD57-** | 30 (19-47) | 29 (15-51) | 32 (14-43) | 0.856 |  |
| **CD4+CD28-CD57+** | 3 (0.4-6) | 3 (0.6-7) | 2 (0.4-4) | 0.310 |  |
| **CD8+CD28-CD57+** | 31 (24-45) | 39 (26-48) | 35 (24-43) | 0.487 |  |
| **CD4+CD28+CD57+** | 2 (1-3) | 3 (1-6) | 2 (0.6-3) | 0.108 |  |
| **CD8+CD28+CD57+** | 2 (1-4) | 3 (2-7) | 2 (1-4) | 0.131 |  |
| **CD4+CD28-CD57-** | 8 (3-13) | 8 (3-10) | 6 (4-22) | 0.948 |  |
| **CD8+CD28-CD57-** | 30 (21-39) | 18 (16-32) | 23 (16-32) | **0.018** | **0.009** nIMT vs iIMT |
|  |  |  |  |  | **0.082**  nIMT vs plaque |

NOTE: Data are shown as median (IQR, Interquartile Range); p* data analyzed by Kruskal-Wallis for comparison between the 3 groups; p** Mann-Whitney test for comparison between 2 groups; IMT: Intima-Media Thickness; nIMT, normal intima-media thickness (IMT) < 1 mm; iIMT, increased IMT (> 1 mm and < 1.5 mm); Plaque, IMT > 1.5 mm at each site or a 50% increase for near-wall thickness > 1.5 mm
